# Two decades of impact: lessons from the HTAi Patient and Citizen Involvement in HTA Interest Group

**DOI:** 10.1017/S0266462326103651

**Published:** 2026-03-05

**Authors:** Aline Silveira Silva, Fiona Pearce, Karen M. Facey, Neil Bertelsen, Janet Wale, Anke-Peggy Holtorf, Ann Single

**Affiliations:** 1 Patient and Citizen Involvement in HTA Interest Group, Canada; 2 University of Brasilia, Brazil; 3 Agency for Care Effectiveness, Government of Singapore Ministry of Health, Singapore; 4 Usher Institute, University of Edinburgh, UK; 5 Neil Bertelsen Consulting, Germany; 6 Cochrane Collaboration, Cochrane Consumer Network, Australia; 7 College of Pharmacy, University of Utah Department of Pharmaceutics, USA; 8 Health Outcomes Strategies GmbH, Switzerland; 9 Patient Voice Initiative, Australia

**Keywords:** health technology assessment, patient involvement, citizen engagement, capacity building, global collaboration

## Abstract

Since its establishment in 2005, the HTAi Patient and Citizen Involvement in HTA Interest Group (PCIG) has worked to strengthen health technology assessment (HTA) by systematically incorporating patient and citizen perspectives. Over two decades, PCIG has advanced this goal through multistakeholder projects and collaborations that have produced practical tools, guidance, and methods to support patient involvement in HTA worldwide. Through global knowledge exchange, PCIG has fostered shared learning and capacity building for inclusive, participatory HTA. Its work continues to drive a shift in the mindset of the HTA community – from passive consultation to active partnership with patients and citizens – and encourages investment in robust research to understand patient perspectives. As health systems increasingly aim to reflect lived experiences and community values to improve the implementation and impact of services, PCIG’s journey offers a compelling example of the long-term value of collective action, sustained engagement, and meaningful involvement in HTA.

## Introduction

HTAi Interest Groups bring together members with shared interests in key HTA-related themes for exchange, collaboration, and the development of methodologies and best practices in HTA (1). The *Patient and Citizen Involvement in HTA Interest Group* (PCIG) is among the most active and long-standing. It has helped shift patient involvement from a peripheral activity to a central consideration in discussions on improving HTA.

This paper marks the 20th anniversary of PCIG. It reflects on PCIG’s evolution, impact, and contributions over the past two decades and highlights key lessons learned and emerging opportunities for the future of patient and citizen involvement in HTA.

Building on the description provided by Facey et al. (2), PCIG defines “patient involvement” as comprising two complementary but equally important approaches: (i) research into patients’ needs, experiences, perspectives, and preferences; and (ii) patient participation across HTA activities, including individual HTAs, methods and process development, and HTA policy. By contrast, “citizen involvement” refers to the use of research and participatory approaches to understand broader societal perspectives from members of the public who are not living with the condition(s) under assessment, nor caring for or supporting someone who is (3). The definition of patient involvement is critical in addressing ongoing confusion among HTA practitioners about the distinct roles and value of both research and participation. This has guided PCIG’s work in developing shared solutions and practical tools that enable effective involvement.

Patient and citizen involvement is widely recognized as essential to fostering HTA decisions that are not only evidence-informed, but also relevant, transparent, accountable, and legitimate (2;4;5;6). This recognition has been hard-won, as clinical and cost-effectiveness data have traditionally dominated when determining the value of a technology in HTA. However, from the early development of HTA as a formal field, there was awareness that assessments should consider wider domains, including impacts on patients and their families (7).

Today, in many jurisdictions, patients and citizens contribute to HTA through direct participation – by taking part in meetings, submitting written input or personal testimonials, and co-designing organizational processes. They also contribute through research that generates evidence on their perspectives, needs, preferences, and lived experiences. Together, these approaches help ensure that HTAs are informed by the people they ultimately serve.

## From connection to lasting collaboration

Drawing on her experience establishing Scotland’s HTA body in 2000, where patient-focused principles were embedded into evidence-informed decision-making, Karen Facey, PCIG’s Founding Chair, connected with like-minded members across the HTAi community to lay the groundwork for a formal network under the HTAi umbrella – marking the beginning of PCIG.

PCIG was born from experience of HTA producers who had involved patients in their HTAs and seen the difference they made to the entire process of an HTA—altering the scope of the HTA, influencing assessment of evidence, to ensuring outputs were accessible. They recognized that patient involvement is complex and a journey that is best travelled collaboratively. – Karen Facey, PCIG Founding Chair (2005–2011)

PCIG’s foundational goals are underpinned by five key values – relevance, fairness, equity, legitimacy, and capacity building (HTAi Values and Quality Standards for Patient Involvement in HTA) – and reflect a commitment to (1;8):

1. *Promote and develop robust methodologies* to incorporate patient and citizen perspectives into HTA, ensuring their experiences, needs, and values are considered alongside clinical and economic evidence.
2. *Strengthen HTA practice* by advocating for the systematic and transparent inclusion of patient and citizen input across all stages of the HTA life cycle.
3. *Share best practices and build global capacity* in developing the skills, tools, and processes needed to elicit, value, and integrate patient and citizen contributions effectively.
4. *Support countries with limited experience* of HTA to elicit and incorporate patient and citizen perspectives.

PCIG began as a small group of dedicated individuals and grew to more than 300 members across 50 countries by 2025, bringing together a diverse community of patient organizations, HTA bodies, industry, academia, independent patient experts, caregivers, and advocates. A Steering Committee representing these perspectives provides strategic direction and oversight, guiding PCIG’s Project Sub-Committees and reporting to the HTAi Board through the Co-Chairs.

## Delivering tools for change and impact

In 2010, three Working Groups were formed to coordinate the growing range of topics PCIG was addressing and actively engage members in core activities:

- *The Methods and Impact Working Group* aimed to identify and promote robust evidence that could influence HTA decision-making by incorporating the views, preferences, and lived experiences of patients and their caregivers.
- *The Patient Involvement and Education Working Group* sought to build knowledge, skills, and opportunities for meaningful patient involvement in HTA globally, with a strong emphasis on capacity building and equity.
- *The Citizen and Community Involvement Working Group* focused on the distinct role of citizen, public, and community perspectives in HTA, exploring mechanisms for engaging broader voices in assessment processes.

These groups provided the foundation for much of PCIG’s early work, creating structured spaces where members from different jurisdictions and stakeholder groups could collaborate to achieve shared goals.

Thanks to its members’ dedication, PCIG has steadily advanced the methodologies, practices, and principles of inclusive HTA. Among its most foundational and influential contributions is a suite of tools and resources developed to support both HTA bodies and patient communities in strengthening or establishing involvement opportunities (Table 1). For example, HTA bodies worldwide have adapted written submission templates for patients to use and have adopted principles from the *Values and Quality Standards* to guide good practice.

**Table 1.** Key tools and resources developed by PCIG (9)

Resource	Description
HTAi Consumer and Patient Glossary (2009)	An introductory guide to key terminology commonly used in HTA, developed to support clearer communication, and help patients and citizens engage in HTA. Patient organizations were invited to use the glossary and provide feedback on its relevance and clarity.
Values and Standards for Patient Involvement (2014)	This Delphi-study-derived tool was co-created with different stakeholders and has been adopted internationally. It is used by HTA bodies and researchers for self-assessment and has been acknowledged, referenced, and, where applicable, translated by HTA agencies in England, Scotland, France, Belgium, Colombia, and Canada.
Guide to Complete Written Submissions and Written Submission Templates (2014)	Recognizing that approaches to involvement vary across jurisdictions, PCIG developed HTA submission templates adaptable to different goals and settings. These have since been translated and tailored for use in various jurisdictions and for different types of health technologies. PCIG also adapted recommendations from the pan-Canadian Oncology Drug Review (PCoDR) to create detailed guidance for patient groups on best practices for collecting and reporting information for input into HTAs.
Ethics Guidance for Patient Organizations (2016)	This guidance, developed with HTAi’s Ethics Interest Group, outlines key ethical considerations for patient organizations collecting and reporting information from patients for HTA submissions, helping ensure transparency, accountability, and integrity in the process.
PARADIGM – Involvement in HTA Early Dialogues: Tools and resources for HTA bodies (2020)	This toolkit is for HTA bodies to adapt and use when engaging patients in Early Dialogue processes. It was co-created through a multistep consultative process led by PCIG as part of the IMI PARADIGM project (Innovative Medicines Initiative) and involved collaborative workshops with HTA bodies, patient organizations, and other stakeholders.
Plain language summaries (2021)	An adaptable template (with guidance) for industry to complete to create a plain language summary of their submissions to HTA for patient groups seeking to provide input to an HTA. The intent is that HTA bodies check the summary to ensure the information is accurate and balanced before sending it to patient organizations.

In 2019, PCIG adopted a project-based structure that enables members to propose and lead initiatives aligned with the group’s strategic goals, with oversight from the PCIG Steering Committee. Since then, numerous projects have been successfully completed or are ongoing, producing outputs that have informed best practices and strengthened the integration of patient and citizen involvement into HTA (Table 2).

*Working on projects allows PCIG members to engage around clearly formulated objectives within a structured project plan, with a defined beginning and end, and provides opportunities to develop, test, and disseminate materials that help address persistent gaps or emerging trends related to patient involvement in HTA. – Anke-Peggy Holtorf, PCIG Project Coordinator (2019–2025)*

**Table 2.** Key PCIG projects, their objectives, and main deliverables

Project title	Objective(s)	Key deliverables
P01: Patient and Citizen Participation at the Organizational Level in HTA	Gain a better understanding of patient and citizen participation in the organizational domain by identifying how, and on what topics, participation is occurring, and the perceptions about this kind of participation.	**Publication:** Identified examples of organizational participation in managerial aspects: governance, defining patient involvement processes, evaluation processes and methods, and capacity building (10)
P02: Stakeholders’ Perspectives of Impact of Patient Involvement in HTA	Gather and analyze multistakeholder perspectives on the perceived impact of patient involvement in a specific HTA to support reflection on the mechanisms, benefits, and burdens of different approaches to involvement.	**Publication:** Identified domains of impact and proposed a practical framework and validated tools that can be used by researchers and HTA bodies to support reporting and increased evaluation in the field (11) **Webinars:** (i) How patient involvement is making a difference in HTA – perspectives of impact (May 2022, 751 views*); (ii) A framework for characterizing impact of patient involvement in HTA (August 2024, 307 views*) (9)
P03: Summary of Information for Patients – SIP Template P10: Implementation of SIP Template (ongoing)	Broaden the adoption of an aligned summary concept and template by HTA bodies; simplify the process for industry to provide this input during their submissions; provide greater information/transparency, and simplify the process for patients to give input during HTAs. P10 is an extension of P03 to pilot the use of the SIP template in select HTA agencies.	**Publications:** (i) Developed an international template to support patient submissions to inform HTAs (12); (ii) Piloted the use of the template in Australia and England to demonstrate the value of plain language summaries for patient organizations intending to provide input to HTAs (13) **Webinar:** Plain language summaries to support patient expert input to HTA (September 2024, 416 views*) (9)
P04: Patient and Citizen Involvement in the Low- and Middle-Income Country (LMIC)	Explore the routes of patient involvement in LMICs; collaborate with LMIC stakeholders to define specific needs for patient involvement; develop and adapt guidance and tools to support patient and citizen involvement in HTA in emerging economies. (Collaboration with the Developing Countries Interest Group of HTAi)	**Publication:** Developed and piloted a descriptive template in four LMIC’s and the emergence of patient and citizen involvement opportunities related to HTA to demonstrate varying stages of participatory HTA or healthcare decision making (14) **Book chapter:** Chapter 28: Perspectives from low- and middle-income countries on patient involvement in HTA co-created with 29 stakeholders from LMICs (15)
P08 and P11: Patient Preferences P13: HTA Deliberations Using Patient Preference Data – A Mock Trial experience with HTA Agencies (ongoing)	Put patient involvement in HTA in the spotlight and provoke debate around the advantages and disadvantages of patient participation versus the development of patient-based evidence in the form of patient preferences; create a clear understanding of the rigor underpinning patient preference studies; provide an opportunity to examine the use of, and potential for patient preference studies to influence HTA. P11 and P13 are extensions of P08 to increase implementation and adoption of patient preferences in decision-making and incorporate quantitative patient preferences into HTA decision-making.	**Publication:** Explored how patient preference data are currently used in HTA by surveying forty respondents from HTA bodies and related organizations across twelve countries. The research highlighted moderate influence of patient preference data on decision-making, stronger perceived value for decision quality, and broad support for patient groups to lead data generation (16) **Webinar:** Do patient preferences influence HTA decisions? (November 2021, 1,200 views*) (9)
P09: Patient Insights Research Platform	Detail the prerequisites and stakeholder expectations for a patient insights research platform as a structured, but purely observatory approach for collecting and analyzing content from patients’ conversations on social media to inform HTA bodies on patient needs and experiences.	**Publications:** (i) Examined in a multi-stakeholder team whether social media research (SMR) addresses current information gaps on patient experiences, preferences, or needs, concluding that SMR can contribute new and relevant evidence to HTA (17); (ii) Conducted a scoping review to distill guidance and academic discourse on ethical or legal issues and standards in using Social Media Research to generate patient insights for HTA or health policy (18)
P12: Patient & Public Involvement at HTAi Annual Meeting	Build on PCIG Participation and Support Scheme (PASS) work to generate guidance to ensure meaningful, welcoming, and well-supported patient and public involvement at HTAi annual meetings.	**Publication:** PCIG recommendations to the HTAi Annual Meeting Committee for patient participation at the HTAi Annual Meetings (9) **Successful participation** of patients in HTAi Annual Meetings: Between 2021 and 2025, 147 patients were supported to join the meeting either in person or virtually (when available)
E002: 360° Patient Involvement in HTA in Europe	Develop a 360° review of current methods and processes for patient involvement in Europe, how they are perceived by those involved, and a co-creation of best practices.	**Publications**: (i) Conducted a structured online survey with 168 HTA stakeholders across 32 European countries to identify good practices and gaps in patient involvement in HTA, revealing consensus on the value of patient insights, and highlighting shortcomings such as lack of systematic processes, limited guidance, and poor communication (19); (ii) 360 report and recommendations (2023; published on Websites: HTAi, EUPATI, and EPF) (9)
P14: Asia Pacific 360: The Practice of Patient Involvement in HTA in the Asia Pacific Region (ongoing)	Provide a living document of the status of patient involvement in HTA in the Asia Pacific region; support patient involvement in HTA in the Asia Pacific region by identifying beliefs about patient involvement in HTA, and actions to support and strengthen existing and new processes; develop or strengthen networks across the region among those working in patient involvement in HTA.	**Webinar:** Patient and citizen involvement in HTA across the Asia Pacific region (November 2024, 300 views*) (9) **Workshops**: Three regional workshops conducted in 2025 with approximately 150 participants

* As of December 2025.

## Capacity building and sharing best practices

A cornerstone of PCIG’s impact is its ongoing commitment to capacity building – empowering patients, researchers, policymakers, and HTA practitioners with the knowledge and skills needed for effective involvement. PCIG delivers webinars in collaboration with international partners, covering topics from foundational principles of involvement to advanced approaches, such as co-production of evidence, ethical considerations, and participatory evaluation (see Table 2). These online sessions are designed to be accessible across time zones and stakeholder groups, enabling broad and inclusive engagement. As of December 2025, they have received almost 3,000 views.

PCIG also organizes interactive workshops for HTAi Annual Meetings and endorses patient-related panel sessions that disseminate study findings, promote discussion, and advance implementation. While involvement practices rightly adapt to the goals and settings of individual HTA bodies, these in-person events encourage shared problem-solving and collective learning. Since the first PCIG workshop in 2005, these sessions have annually brought together between 40 and 70 participants, including patients, caregivers, representatives from patient organizations, HTA agencies, industry sponsors, and other stakeholders seeking to understand how to initiate or evolve patient involvement in HTA. When COVID-19 prevented face-to-face interactions, online exchanges were held under the Chatham House rule (when a meeting, or part thereof, is held under the Chatham House Rule, participants are free to use the information received, but neither the identity nor the affiliation of the speaker(s), nor that of any other participant, may be revealed) were held to provide peer-to-peer learning. Beyond the conference setting, PCIG members also deliver training in their own jurisdictions and regions, tailoring content to local needs and levels of HTA maturity. To strengthen ongoing member engagement, PCIG produces a monthly e-bulletin featuring new resources, upcoming events, and collaboration opportunities. Together, these activities foster continuous knowledge exchange and sustain momentum across PCIG’s international network.

In 2021, PCIG launched the Participation and Support Scheme (PASS) to provide financial assistance, sponsored by health technology developers, for patients, caregivers, and patient representatives to attend the HTAi Annual Meeting and share their expertise, while building their knowledge and networks. A notable outcome of these patient interactions is the Patient Exchange Platform, established in 2025 by former PASS recipients to connect patient representatives involved in HTA around the world, fostering shared learning, and exchange of challenges and best practices across jurisdictions via online meetings.

*Key to delivering practical outputs to support uptake and good practice in patient involvement, is our genuine approach to multi-stakeholder working which always includes partnering with patients and their organizations. By attracting sponsorship for our patient grant and support program, we’ve been able to extend the influence of patient members beyond PCIG projects to contributing to every aspect of HTAi’s Annual Meeting, including planning, leading abstracts and plenary presentations. This has made patient involvement a critical focus for HTAi and seen other Interest Groups involve patients and benefit from patient knowledge. – Ann Single, PCIG Chair (2019–2023)*

All these opportunities mentioned above are especially valuable for jurisdictions with limited experience in patient involvement and early-career researchers, as the concept of involvement can be both inspiring and complex. PCIG challenges traditional assumptions about expertise and encourages reflection on patient knowledge, ethics, evidence, and impact even for well-established HTA bodies.

PCIG’s work over the past two decades offers a rich source of learning for those seeking to embed participation practices and research methods in their HTA processes. Notably, many PCIG members contributed to both editions of *Patient Involvement in HTA*, the first textbook on this subject. The first edition was launched at a full-day workshop at the HTAi 2017 Annual Meeting, with the second edition published as an open-access resource in 2026 (15).

*Capacity building is more than training. It is creating a shared sense of understanding and purpose on the difficult challenges that HTA must balance and building skills as well as knowledge on how this balance can be better navigated with patient and citizen involvement. PCIG has empowered communities to work together to enact meaningful change and through this, has facilitated trust between the main HTA stakeholders. – Neil Bertelsen, PCIG Chair (2016–2019)*

PCIG has played a central role in elevating the global conversation on the importance of patient and citizen involvement in HTA through strategic partnerships, thought leadership, and active engagement in international forums. This commitment has also contributed to a growing body of publications that expand the evidence base on its impact and methodologies. Among PCIG’s most seminal contributions is the paper by Facey et al. (2), which defined patient involvement in HTA and remains the most frequently cited work in this field (20;21).

PCIG members have contributed to different international HTA networks, including EUPATI (European Patients’ Academy on Therapeutic Innovation), EUnetHTA (European Network for HTA), Guidelines International Network (GIN), and the International Network of Agencies for Health Technology Assessment (INAHTA; 22;23), to help embed patient and citizen involvement into practices and guidance.

## Looking ahead: sustaining and evolving patient involvement

PCIG aims to deepen its influence to embed patient and citizen perspectives more meaningfully in HTA systems worldwide by supporting HTA bodies to build on participation with robust evidence of needs, preferences, experiences, and perspectives, and implement processes and structures better able to act on patient knowledge. Early PCIG work highlighted the need for acknowledging the impact of patient and caregiver input on HTA deliberations (24). More recent work has expanded this understanding, categorizing the impact of patient involvement not only on HTA outcomes and recommendations but also on the HTA organizations themselves and on the patients involved (11).

For me, patient involvement in HTA has never been about checking a box or meeting a mandate—it’s about shifting mindsets to genuinely value lived experiences as part of the evidence base. I’ve seen how meaningful involvement can enrich not only the decisions we make, but also the people, processes, and relationships behind them. When done intentionally and well, it brings legitimacy, humanity, and clarity to complex choices. – Aline Silva, PCIG Co-Chair (2023-present)

Health systems evolve, so too must the approaches used to involve patients and citizens in HTA. Sustaining this progress requires a continued focus on robust, up-to-date methods, capacity building, and evaluation of quality and impact. PCIG’s work can help HTA bodies move beyond measuring participation rates to understanding how involvement influences legitimacy, trust, and decision-making. Through partnerships with academics and HTA bodies, PCIG will continue to develop practical, evidence-informed approaches that strengthen the inclusion of patient and citizen perspectives across HTA systems and ensure that efforts are purposeful and effective.

*PCIG has always been driven by the belief that better health decisions are made when the people affected by them have a seat at the table. As we look ahead, our focus is on building stronger regional connections, supporting inclusive practices, and ensuring our work continues to evolve with the needs of the communities we serve. – Fiona Pearce, PCIG Co-Chair (2023-present)*

## Global equity as a priority

Many low- and middle-income countries (LMICs) are still in the early stages of integrating involvement practices in HTA. PCIG can support these efforts by sharing adaptable tools, fostering peer learning and building partnerships that reflect local values and contexts. A more equitable HTA ecosystem requires amplifying diverse voices and acknowledging that “best practice” must be flexible, culturally responsive, and tailored to different settings. In collaboration with the HTAi Developing Countries Interest Group, a project subcommittee representing these regions continues to identify good practices and explore opportunities to strengthen involvement in these contexts (14).

*One of PCIG’s greatest strengths is our ability to connect people across borders. We’re fostering a truly global conversation about what meaningful involvement looks like, and how systems—regardless of context—can learn from each other to build more inclusive approaches to HTA. – Janney Wale, PCIG Chair (2011–2016)*

Finally, the future of patient and citizen involvement relies on overcoming resourcing limitations and having resilient networks – communities that can adapt, learn from shortcomings, and continue to evolve. PCIG is committed to being such a network: grounded in its standards and values, shaped by diverse voices, and open to how it meets its goals.

## Conclusion

As PCIG enters its third decade, its legacy is marked not just by tools and frameworks but by the passion, perseverance, and leadership of those who have steered its course. What began as a small group advocating for the inclusion of patient and citizen voices in HTA has evolved into a global network shaping how health technologies are assessed, valued, and understood.

Over the years, PCIG has demonstrated that involvement is not a static goal but a dynamic practice – one that must continually adapt to new contexts, challenges, and opportunities. Its impact is evident in the growing recognition of lived experience as essential to legitimate and accountable decision-making in healthcare, and in sustained efforts to build capacity, foster collaboration, and influence policy across the globe.

The work is ongoing. Ensuring equitable, inclusive, and impactful involvement in HTA requires continued commitment, innovation, and investment. As HTA systems become more complex, PCIG is uniquely positioned to champion inclusive practices based on strong and robust but also efficient patient and citizen involvement in HTA. Its efforts to support newcomers, assess quality and impact, support and nurture the next generation of engaged HTA communities, as well as continuing to normalize patient perspectives in its own work, will be central to its future.

PCIG’s enduring strength lies in its community – a diverse, global collective united by a shared belief that health decisions are better when they include the people most affected by them. That belief will continue to guide PCIG’s work in the years ahead as it builds on its foundations and leads the way toward more responsive, inclusive, and participatory HTA globally.
